# Long Non-coding RNAs Are Differentially Expressed After Different Exercise Training Programs

**DOI:** 10.3389/fphys.2020.567614

**Published:** 2020-09-15

**Authors:** Bernardo Bonilauri, Bruno Dallagiovanna

**Affiliations:** Laboratory of Basic Biology of Stem Cells (LABCET), Carlos Chagas Institute – FIOCRUZ-PR, Curitiba, Brazil

**Keywords:** lncRNAs, exercise, training, RNA-seq, muscle, HIIT

## Abstract

**Background:**

Molecular regulation related to the health benefits of different exercise modes remains unclear. Long non-coding RNAs (lncRNAs) have emerged as an RNA class with regulatory functions in health and diseases. Here, we analyzed the expression of lncRNAs after different exercise training programs and their possible modes of action related to physical exercise adaptations.

**Methods:**

Public high-throughput RNA-seq data (skeletal muscle biopsies) were downloaded, and bioinformatics analysis was performed. We primarily analyzed data reports of 12 weeks of resistance training (RT), high-intensity interval training (HIIT), and combined (CT) exercise training. In addition, we analyzed data from 8 weeks of endurance training (ET). Differential expression analysis of lncRNAs was performed, and an adjusted *P*-value < 0.1 and log2 (fold change) ≥0.5 or ≤−0.5 were set as the cutoff values to identify differentially expressed lncRNAs (DELs).

**Results:**

We identified 204 DELs after 12 weeks of HIIT, 43 DELs after RT, and 15 DELs after CT. Moreover, 52 lncRNAs were differentially expressed after 8 weeks of ET. The lncRNA expression pattern after physical exercise was very specific, with distinct expression profiles for the different training programs, where few lncRNAs were common among the exercise types. LncRNAs may regulate molecular responses to exercise, such as collagen fibril organization, extracellular matrix organization, myoblast and plasma membrane fusion, skeletal muscle contraction, synaptic transmission, PI3K and TORC regulation, autophagy, and angiogenesis.

**Conclusion:**

For the first time, we show that lncRNAs are differentially expressed in skeletal muscle after different physical exercise programs, and these lncRNAs may act in various biological processes related to physical activity adaptations.

## Introduction

Despite the application of molecular techniques to studies on exercise biology, the molecular landscape of physical exercise-induced regulation is poorly understood. Physical activity results in characteristic molecular responses at the DNA, RNA, and protein levels (Booth et al., 2002; Grazioli et al., 2017). Endurance and high-intensity interval training (HIIT) increase cardiovascular fitness by increasing VO_2_max, resulting in increased physical capacity and decreased mortality rates. One molecular pathway for these exercise adaptations is through transcriptional regulation of PGC1-α, which has direct effects on mitochondrial biogenesis, fiber type specification, angiogenesis, and GLUT4 regulation in skeletal muscle (Lira et al., 2010; Torma et al., 2019). On the other hand, resistance exercise (RT) is known to activate the mTOR pathway, resulting in the synthesis of myofibrillar proteins causing increased muscle strength and muscle hypertrophy (Song et al., 2017). In any case, exercise training improves muscle function and overall health and is an important tool to prevent and treat several common diseases.

Recently, long non-coding RNAs (lncRNAs) emerged as an RNA class with regulatory functions. These RNAs are commonly described as sequences longer than 200 nucleotides that lack the ability to encode proteins and have high tissue-specific expression – more so than protein-coding genes. LncRNAs are involved in the regulation of cell growth and differentiation, transcription, splicing, translation, X chromosome inactivation, myoblast proliferation and muscle development (Mercer et al., 2009; Cabili et al., 2011; Ma et al., 2013; Sweta et al., 2019). For example, lncRNA PVT1 impacts mitochondrial respiration, apoptosis and myofiber size in mouse models of muscle atrophy (Alessio et al., 2019). Myoregulin (MRLN), a newly discovered micropeptide derived from the small ORF (sORF) of LINC00948, interacts with SERCA in the membrane of the sarcoplasmic reticulum and regulates Ca2+ handling. When wild-type and MRLN KO mice were subjected to a regimen of forced treadmill running to exhaustion, the MRLN KO mice presented a 55% increase in running distance, thus improving muscle performance (Anderson et al., 2015). LncRNAs can also function as competing endogenous RNAs (ceRNAs), binding and sequestering complementary microRNAs preventing their inhibitory function on mRNAs and, thus, having a direct impact on gene expression in skeletal muscle (Cesana et al., 2011; Li H. et al., 2018). Recently, microarray analysis showed that lncRNAs were differentially expressed in the aortic endothelium after long-term exercise training in mice with insulin resistance (Liu et al., 2018).

Second-generation sequencing technologies, such as RNA-seq, have demonstrated extraordinary analytical potential and have been used in molecular biology and lncRNA expression analysis studies (Wang et al., 2009; Dallagiovanna et al., 2017). The ease-of-use of this technology to study lncRNAs expression is due to the similar features between lncRNAs and protein-coding genes. They are transcribed by RNA polymerase II into spliced or unspliced 5′-capped and 3′-polyadenylated RNAs (Quinn and Chang, 2016). Unfortunately, sports medicine has not kept pace with advances in genomics, transcriptomics and proteomics, with only a few studies published in this field (Zierath and Wallberg-Henriksson, 2015; Pourteymour et al., 2017; Robinson et al., 2017; Popov et al., 2019; Pillon et al., 2020). To date, no studies have been conducted using RNA-seq data to investigate lncRNA expression in skeletal muscle after different types of physical activities. Here, we demonstrated that lncRNAs are differentially expressed in skeletal muscle after different physical training protocols, and this expression is exercise specific.

## Materials and Methods

### Data and Study Overview

The current study was conducted using the data from Robinson et al. (2017) and Popov et al. (2019) collected from untrained young individuals (males and females among 18–30 years and males among 21–24 years, respectively) subjected to different exercise programs. Exercise training protocols consisted of resistance training (RT) of upper and lower body exercises (4 sets of 8–12 repetitions with 1-min rest between sets) 2 days each per week. Upper body exercises were: lat pull down, incline chest press, chest press, seated row, lateral raise, biceps curl, and triceps push down. Lower body exercises were: leg press, toe raise, lunge, abdominal crunch, leg extension, and leg curl. HIIT consisted of 3 days per week of cycling (4 cycles of 4 min at >90% of peak oxygen consumption [VO_2_ peak] with 3 min pedaling at no load) and 2 days per week of treadmill walking (45 min at incline at 70% VO_2_ peak). Combined training (CT) consisted of 4 days per week of weight lifting with fewer repetitions than RT and 5 days per week of cycling (30 min at 70% VO_2_ peak). For endurance training (ET), subjects used a two-legged cycle ergometer for 8 weeks (60 min/day, 5 days per week). The aerobic training program consisted of 60 min of continuous cycling (70% blood lactate concentration of 4 mmol/l [LT_4_]) and intermittent cycling (3 min, 50% LT_4_ + 2 min, 80% LT_4_ × 12).

### Collection of High-Throughput Datasets and Bioinformatics Analysis

Raw data (RNA-seq) were downloaded from the Gene Expression Omnibus (GEO) database^1^ under the accession numbers GSE97084 and GSE120862. Briefly, we selected 63 fastq files from young subjects, which included 22 fastq files from HIIT (11 baseline and 11 post training), 20 fastq files from RT (10 baseline and 10 post training), 15 fastq files from CT (8 baseline and 7 post training) and 6 fastq files from ET (3 baseline and 3 post training). For each training program, subjects are their own control group.

The RNA-seq data were first subjected to a quality control check with FastQC (v.0.11.2). Raw reads were searched for rRNA sequences using Bowtie2 (v.2.2.5). Then, the remaining reads were mapped to genome build hg19 (GRCh37) using STAR (v.2.5.3a). Only unique mapped reads were selected, and sample principal component analysis was verified (Supplementary Figure S1). RT samples had an average of approximately 23,7 million reads (total of 475,786,397 reads), with 92,79% mapped reads. CT samples had an average of approximately 23,5 million reads (total of 353,831,878 reads), with 93.23% mapped reads. HIIT samples had an average of approximately 23,1 million reads (total of 509,854,713 reads), with 92,78% mapped reads. ET samples had an average of approximately 52,6 million reads (total of 315,875,344), with 90,3% mapped reads. featureCounts (v.1.6.0) was used to count the read numbers mapped to known genes using gene annotation GTF files from GENCODE (v.31). Differential gene expression analysis was performed using DESeq2 (v.1.20.0) from raw counts. We selected cutoff criteria of log2 (fold change) ≥0.5 or ≤−0.5 and adjusted *P*-value < 0.1. Statistical and other analyses were performed using R (v.3.5.2). For coding probability calculation, we selected DELs sequences in FASTA format and performed CPC2 analysis. CPC2 identified four intrinsic features: Fickett Score, ORF length, ORF integrity and isoelectric point (pI), being the Fickett Score derived from weighted nucleotide frequency of the full length transcript and the remaining features are *in silico* calculated based on the longest putative ORF (Kang et al., 2017).

### Coexpression Network Analysis of lncRNAs

To identify the potential pathways and functions of lncRNA action, CEMiTools (Russo et al., 2018) was used to automatically generate a gene coexpression network and perform pathway enrichment using Gene Ontology (GO) from EnrichR tool (Kuleshov et al., 2016) with “GO Biological Process 2018” database and *P*-value ≤ 0.01. Briefly, CEMiTools uses an unsupervised filtering method based on the inverse gamma distribution for gene selection. After that, a soft threshold power β is chosen using a modified algorithm and this value is used to determine a similarity criterion between pairs of genes (Russo et al., 2018). The identified modules were filtered based on the presence of differentially expressed lncRNAs in clusters.

### ceRNA Network Prediction

LncRNA-microRNA interaction predictions were performed using AnnoLnc. In briefly, AnnoLnc calculates the conservation score of interactions in primate, mammals and vertebrates clade (Hou et al., 2016). From this score, we are able to identify miRNAs with greater confidence in addition to search them in the literature related to skeletal muscle biology and/or physical exercise. After miRNAs identification, all predicted miRNA-target mRNAs were collected from miRBase (Kozomara and Griffiths-Jones, 2011), and one miRNA was selected to develop a lncRNA-miRNA-mRNA network. For this, predicted target mRNAs were compared to differentially expressed up-regulated protein-coding genes after HIIT, and from these, we performed a Gene Ontology (GO) analysis. The network was created in Cytoscape (v.3.5.0).

## Results

### LncRNAs Are Differentially Expressed After Different Physical Training Programs

**FIGURE 1:**
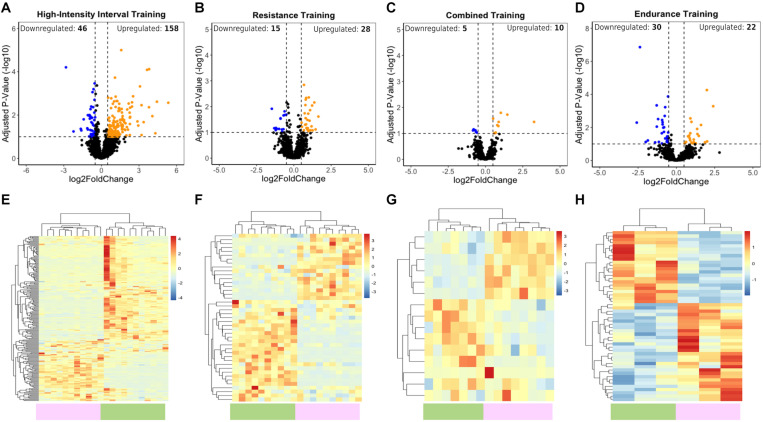
LncRNA transcriptome profiles associated with HIIT, RT, CT, and ET exercises. **(A–D)** Volcano plots show upregulated (orange) and downregulated (blue) DELs. Vertical dotted lines represent the log2-fold change cutoff (right and left), and values above the horizontal dotted lines represent lncRNAs with adjusted *P*-value < 0.1. **(E–H)** Heatmaps showing normalized differentially expressed lncRNAs between the posttraining (green bar) and baseline (purple bar) samples after HIIT, RT, CT, and ET. Blue indicates low lncRNA expression, and red indicates high lncRNA expression.

To explore lncRNA expression after different exercise programs, we performed bioinformatics analysis of RNA sequencing (RNA-seq) data from baseline and post exercise skeletal muscle biopsies of young subjects (see section “Materials and Methods”). We identified significantly distinct patterns of lncRNA expression related to different types of physical exercise. Using ≥0.5 and ≤−0.5 Log2 (fold change), and 10% adjusted *P*-value cutoff criteria, we identified a total of 204 lncRNAs differentially expressed after 12 weeks of HIIT (158 lncRNAs upregulated and 46 downregulated) (Figures 1A,E), including the muscle-related lncRNA LINCMD1. After RT, a total of 43 lncRNAs were differentially expressed (28 lncRNAs upregulated and 15 downregulated) (Figures 1B,F). For CT, a total of 15 lncRNAs were differentially expressed (10 lncRNAs upregulated and five downregulated) (Figures 1C,G). In the ET group, we identified a total of 52 lncRNAs that were differentially expressed, with 22 upregulated and 30 downregulated (Figures 1D,H) (Supplementary Tables S1–S4). These findings indicated a differential expression profile of lncRNAs during different exercise training programs.

### Differences in lncRNA Expression Among Different Physical Training Programs

**FIGURE 2:**
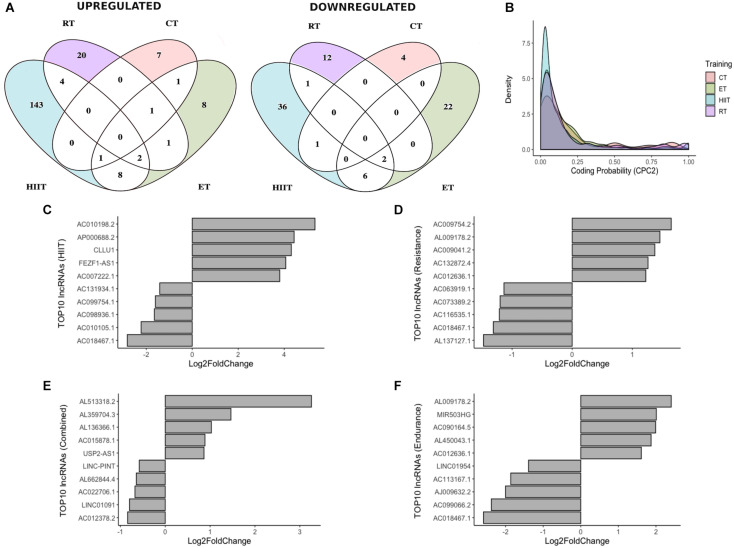
Differentially expressed lncRNAs in different physical training programs. **(A)** Venn diagram showing differentially expressed lncRNAs upregulated and downregulated during different training modes. **(B)** Density plot showing the coding potential probability of lncRNAs, confirming their non-coding capacity. **(C–E)** Expression status of the top 5 upregulated and top 5 downregulated lncRNAs after 12 weeks of HIIT, RT, or CT and after 8 weeks of ET **(F)**.

For a better understanding of lncRNA expression regulation after HIIT, RT CT, and ET, we proceeded with pairwise comparisons (HIIT/RT, HIIT/CT, HIIT/ET, RT/CT, RT/ET, CT/ET). The four physical training programs showed highly specific and distinct lncRNA expression profiles and clustered separately, with only a few common lncRNAs (Figure 2A). DELs were tested for their coding potential capacity using CPC2 to ensure that the obtained lncRNAs do not encode proteins (Figure 2B), and the top 10 DELs (five upregulated and five downregulated) in each training program are shown (Figures 2C–F). All DELs were manually and individually researched in the literature, with approximately 23% of total identified lncRNAs with at least one publication record (data not shown). Most studies were related to cancer research, with few studies related to the physiological effects of these ncRNAs.

### Potential Functional Characterization of DE lncRNAs Using Coexpression Network Analysis

Despite recent efforts to characterize lncRNA mechanisms of action, most lncRNAs have unknown functions. We used coexpression network analysis to infer the potential pathways of action of DELs. First, just to have a broader view, we performed GO analysis of differentially expressed protein-coding genes for each training program (Supplementary Tables S5–S8). We identified the same biological processes reported by Robinson et al. For instance, after 12 weeks of HIIT, biological processes related to extracellular matrix organization, collagen fibril organization, protein complex subunit organization and regulation of angiogenesis were observed. Similarly, we also observed for RT biological processes related to extracellular matrix organization, regulation of angiogenesis, regulation of cell migration, regulation of PI3K and regulation of intracellular signal transduction.

**FIGURE 3:**
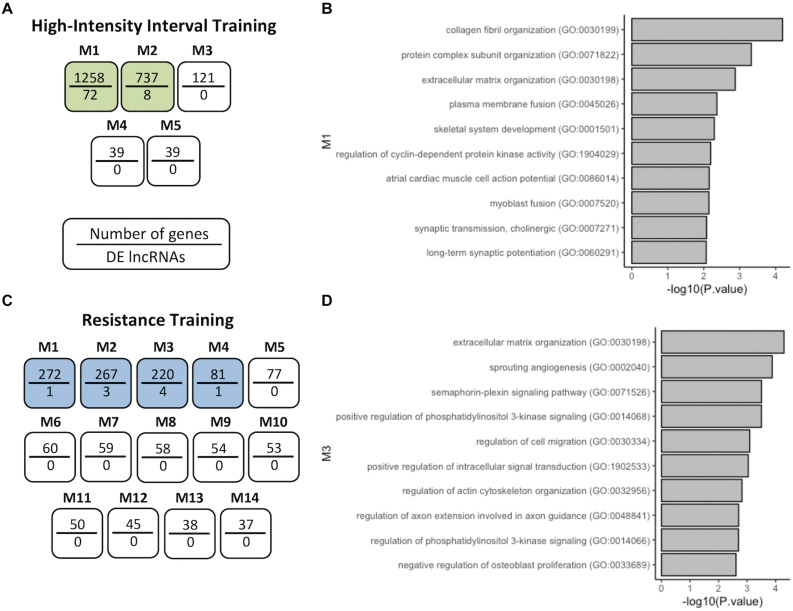
Coexpression network analysis of genes in HIIT and RT. **(A)** HIIT modules after CEMiTool analysis showing that M1 and M2 coexpressed DELs. **(B)** GO terms of coexpressed protein-coding genes of M1-HIIT. **(C)** RT modules M1 to M4 coexpressed DELs. **(D)** GO terms of coexpressed protein-coding genes of M3-RT. The modules with at least one DEL are analyzed, shown here as modules with a higher number of DELs.

Following this, we used a coexpression module identification tool (CEMiTool) to group DELs into different functional modules (Figure 3). For HIIT, 5 modules with only two modules containing DELs were observed (Figure 3A). For RT, 14 modules with only four modules with at least one DEL were observed (Figure 3C). For CT and ET, no module was observed to contain DELs. Pathway analysis (Gene Ontology) of the modules with DELs was conducted with EnrichR (see section “Materials and Methods”). In module 1 of HIIT, we observed 72 DELs (Supplementary Table S9), and GO analysis of the coexpressed protein-coding genes demonstrated possible functions related to collagen fibril organization, protein complex subunit organization, extracellular matrix (ECM) organization, CDK regulation, myoblast and plasma fusion, skeletal system development and synaptic transmission (Figure 3B). In module three of RT, we observed four DELs (Supplementary Table S10), and GO analysis demonstrated possible functions related to extracellular matrix organization, sprouting angiogenesis, semaphorin-plexin signaling pathway, positive regulation of PI3K, regulation of migration, positive regulation of actin cytoskeleton organization and regulation of intracellular signal transduction (Figure 3D). Module 2 of HIIT and modules 1, 2, and 4 of RT are shown in Supplementary Figure S2, demonstrating possible functions related to the regulation of receptor binding, actin-myosin filament sliding and striated muscle contraction, for example.

### Competitive Endogenous RNAs in Exercise Physiology

**FIGURE 4:**
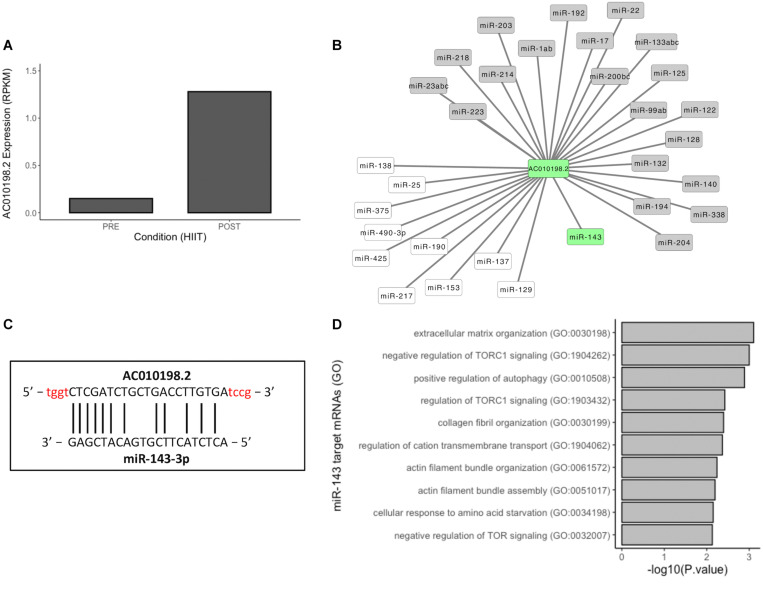
Competitive endogenous RNA network of lncRNAs/miRNAs. **(A)** Expression profile of AC010198.2 in baseline and posttraining. **(B)** Predicted lncRNA-miRNA interaction network of AC010198.2. miRNAs in gray boxes are documented to have muscle functions. **(C)** Binding site of AC010198.2 and miR-143-3p, and **(D)** top 10 terms of GO analysis of miR-143 upregulated target mRNAs after 12 weeks of HIIT.

Long non-coding RNAs have the ability to act as competitive endogenous RNAs (ceRNAs) to sponge and suppress the activity of bound miRNAs, effectively de-repressing other targets of these miRNAs (Thomson and Dinger, 2016). Here, we selected lncRNA AC010198.2 (top upregulated after HIIT) (Figures 2C and 4A) to investigate the interaction network of this lncRNA with miRNAs. Prediction interaction analysis identified 31 miRNAs/miRNA families, including 21 known skeletal muscle regulatory miRNAs in vertebrates (e.g., miR-1 and miR-133) (Figure 4B) (Honardoost et al., 2015; Lozano-Velasco et al., 2015; Zhao et al., 2016; Li et al., 2017, p. 223; Cerro-Herreros et al., 2018; D’Agostino et al., 2018; Siracusa et al., 2018; Wang et al., 2018; Kappeler et al., 2019; Kong et al., 2019). We highlighted miR-143, which has one binding site to AC010198.2 (Figure 4C), and then we analyzed the predicted mRNAs targets. Thus, 1176 target mRNAs were identified and compared to differentially expressed (upregulated) mRNAs after HIIT, with 63 mRNAs being identified. Gene Ontology (GO) analysis of these mRNAs is represented in Figure 4D. LncRNA AC010198.2 may act by sponging miR-143, thus regulating pathways such as positive regulation of autophagy, TOR regulation, and actin filament bundle assembly and organization. In conclusion, lncRNAs upregulated after different exercise programs may act as ceRNAs, regulating miRNA activity in skeletal muscle and releasing mRNAs from inhibition to allow translation.

## Discussion

It is well known that the initiation of an exercise program elicits molecular adaptations to stimuli, but until now, no data regarding skeletal muscle lncRNA differential expression after exercise have been reported. Here, we described the differential expression analysis of lncRNAs after different physical training programs and the potential pathways and mechanisms of lncRNA action related to physiological responses to exercise.

### Long Non-coding RNAs: Possible Pathways of Action in Response to Exercise

Coexpression network analysis identified possible pathways of action of certain lncRNAs after HIIT and RT. Gene Ontology (GO) analysis revealed pathways related to collagen fibril organization, ECM organization, protein complex subunit organization, synaptic transmission (cholinergic), long-term synaptic potentiation, regulation of CDK activity, skeletal system development, and plasma and myoblast fusion after 12 weeks of HIIT. Recently, an increase in myonuclear numbers at stages of HIIT has been demonstrated in mice, indicating that myoblast fusion occurs during this exercise regimen, and this process is important for exercise adaptations (Goh et al., 2019). Healthy young females submitted to 12 weeks of HIIT showed increased lean body mass and bone mineral content, demonstrating the effect of HIIT on the musculoskeletal system (Brown et al., 2018). The effects on the neuromuscular system are distinct from those of endurance training; HIIT increased motor unit discharge and maximal voluntary contractions (Martinez-Valdes et al., 2017).

GO analysis of the RT coexpression network showed pathways related to ECM organization, sprouting angiogenesis, positive regulation of PI3K, regulation of actin cytoskeleton organization, semaphorin-plexin signaling, regulation of axon extension involved in axon guidance, actin-myosin filament sliding, striated muscle contraction, positive regulation of intracellular signal transduction, and regulation of osteoblast proliferation. In agreement with our results, it has been shown that expansion of the skeletal muscle microvascular network after 12 weeks of RT, skeletal muscle capillarization, and angiogenesis-related pathways were increased in young trained men (Holloway et al., 2018). Resistance training is also able to increase the number of myosin/actin filaments inside sarcomeres, promoting myofibrillar hypertrophy in skeletal muscle and improving myofiber innervation (Wilborn and Willoughby, 2004; Messi et al., 2016). These findings corroborate our analysis and shed light on the possible molecular mechanisms of physical training adaptations, suggesting that lncRNAs may regulate these important pathways in exercise physiology.

### Long Non-coding RNAs: Possible Functions in Skeletal Muscle

Hundreds of lncRNAs have no documented functions yet, and several hypotheses have been proposed for their possible functions. Competitive endogenous RNA and transcriptional regulation mechanisms have emerged as possibilities (Thomson and Dinger, 2016). For example, one of the most studied lncRNAs, lncRNA H19, was found to be upregulated after HIIT and ET in this study (Log2FC = 0.79 and 0.86, respectively). H19 is significantly decreased in the muscle of humans with type-2 diabetes and insulin-resistant rodents, and this decrease is correlated with increased let-7 bioavailability. H19 may regulate muscle glucose metabolism by acting as a ceRNA and sponging let-7 (Gao et al., 2014). Another possible pathway of action of H19 in increasing insulin sensitivity is by activating AMPK in skeletal muscle. Interestingly, a high-fat diet downregulates muscle H19 in mice, and a high-fat diet and inflammation are key contributors to type-2 diabetes and insulin resistance (Duan et al., 2018; Geng et al., 2018). HIIT improved glucose metabolism by mechanisms independent of mitochondrial adaptations in diabetic mice (Chavanelle et al., 2017) with H19 regulation being a possible mechanism. Furthermore, H19 is also a host gene for miR-675, whereby knockdown of H19 RNA in myoblast cells (human and murine) decreases skeletal muscle differentiation, and this inhibition is rescued by the exogenous expression of miR-675 (Dey et al., 2014). Moreover, when myoblasts derived from patients with Duchenne muscular dystrophy (DMD) were treated with synthetic preimplantation factor (sPIF), myoblasts differentiated. The effects were mediated through increased expression of utrophin (homolog of dystrophin), and this effect was mediated via the upregulation of H19/miR-675 and downregulation of let-7 (Morgoulis et al., 2019). DMD-induced pluripotent stem cells (DMD-iPSCs) subjected to skeletal muscle differentiation at day 10 showed downregulation of several somite markers, including H19 (Mournetas et al., 2019). Another muscle-related lncRNA is LINCMD1, which plays a role in muscle differentiation by acting as a ceRNA and sponging miR-133/miR-135. When overexpressed or downregulated, LINCMD1 promotes or inhibits the muscle differentiation program in myoblasts, respectively. In addition, LINCMD1 expression is markedly reduced in muscle cells from DMD patients (Cesana et al., 2011; Legnini et al., 2014). In a mouse model of muscle contusion, a significant increase in lncRNAs, including lincMD1 and H19, was observed, indicating the possible contribution of lncRNAs to the regeneration of skeletal muscle after contusion injury (Zheng et al., 2019). In the current study, we determined that LINCMD1 was upregulated after 12 weeks of HIIT (log2FC = 1.8).

Thereafter, we constructed an *in silico* lncRNA-miRNA prediction network of lncRNA AC010198.2, which was upregulated after HIIT (log2FC = 5.33). At the time of this publication, no literature records have been found regarding this lncRNA. The lncRNA-miRNA network revealed a possible interaction with 31 miRNAs, including MyomiRs, miR-1, and miR-133 (Siracusa et al., 2018). We selected miR-143 for further analysis because little is known about its regulation in human skeletal muscle. Human adult dental pulp stem cells subjected to myogenic differentiation *in vitro* showed a significant decrease in miR-143 expression, and this downregulation was related to apparent myocytic properties (Li et al., 2015). In porcine muscle, miR-143 may be involved in fiber type regulation when downregulated in satellite cells to induce a reduction of the slow muscle fiber gene and protein MYH7, indicating a possible role in the regulation of slow muscle fiber differentiation (Zuo et al., 2015). In bovine muscle satellite cells, downregulation of miR-143 promoted an increase in the muscle differentiation process and inhibited cell proliferation (Zhang et al., 2017). All possible miR-143 mRNA targets were selected and then compared with upregulated DE mRNAs in the HIIT group. Gene Ontology analysis showed pathways related to ECM organization, negative regulation of TORC1 signaling, positive regulation of autophagy, collagen fibril organization, regulation of cation transmembrane transport and actin filament organization and assembly. HIIT promotes improved physical performance through different pathways, including increasing the fiber type area in fast twitch fibers and basal autophagic activities. Autophagy is negatively regulated by target of rapamycin complex 1 (TORC1) kinase, and inhibition of TORC1 enhances the lifespan (Li F.-H. et al., 2018; Escobar et al., 2019; Torma et al., 2019). In summary, lncRNAs may act as ceRNAs in skeletal muscle in response to different physical exercises stimuli.

Little is known about the dynamics of lncRNA expression after physical exercise. Here, we described for the first time the differential expression of lncRNAs in skeletal muscle after four different training programs. Despite these initial findings presented here, further studies should be conducted for a deeper comprehension of lncRNA expression and function related to physical training stimulation and adaptation. Some important issues must be addressed that may influence and biased the observed results. So far, it is unknown how group composition i.e., number of participants, sex, age, weight, BMI, physical capacity, among others, and the different training protocols considering e.g., stimuli, intensity, time between series and time under tension could affect lncRNA expression in skeletal muscle.

### Perspectives

Therapeutic targeting of lncRNAs has been poorly investigated thus far. The modulation of lncRNAs can be performed by chemical compounds, gene therapy or through different interventions (Wan et al., 2017; Saghafi et al., 2019). Here, we shed light on another possible strategy for lncRNA modulation through physical exercise training programs to improve health and performance or for the treatment of different conditions, such as type-2 diabetes, metabolic diseases, muscle diseases, neurodegenerative diseases and cancer. Further studies are needed for a more in-depth investigation on the expression of lncRNAs in different pathologies and how physical exercise intervention can modulate and modify the expression of disease-related lncRNAs.

## Conclusion

By combining different bioinformatics tools and analyses of RNA-seq data from skeletal muscle biopsies of young subjects undergoing different physical training programs, our study demonstrated that long non-coding RNAs (lncRNAs) are differentially expressed after high-intensity interval training, resistance training, combined training and endurance training. LncRNA expression proved to be exercise-specific, and few lncRNAs were commonly expressed between the exercise modes. We identified 204 differentially expressed lncRNAs (DELs) after the HIIT program, 43 DELs after the RT program, 15 DELs after the CT program and 52 DELs after the ET program.

## Data Availability Statement

Publicly available datasets were analyzed in this study. Raw data (RNA-seq) were downloaded from the Gene Expression Omnibus (GEO) database (www.ncbi.nlm.nih.gov/geo) under the accession numbers GSE97084 and GSE120862.

## Author Contributions

BB contributed to the conception and design of the study, analysis and interpretation of the data, and drafting, writing, and revising of the manuscript. BD has substantial contributions to manuscript revision and content debate. Both authors contributed to the article and approved the submitted version.

## Conflict of Interest

The authors declare that the research was conducted in the absence of any commercial or financial relationships that could be construed as a potential conflict of interest.

## Footnotes

1 www.ncbi.nlm.nih.gov/geo
