# Supplementary material for: Long Non-coding RNAs Are Differentially Expressed After Different Exercise Training Programs
Source: Front Physiol. 2020 Sep 15;11:567614. doi: 10.3389/fphys.2020.567614 (PMC7533564; doi:10.3389/fphys.2020.567614)
Supplement: Supplementary file 1 [file Table_1.DOCX]

**Supplementary Files**

**Long Noncoding RNAs are Differentially Expressed after Different Exercise Training Types**

Bernardo Bonilauri^1^*, Bruno Dallagiovanna^1^

^1^ Laboratory of Basic Biology of Stem Cells (LABCET), Carlos Chagas Institute - FIOCRUZ-PR, Curitiba, Paraná, 81830-010, Brazil.

*To whom correspondence should be addressed at: Laboratory of Basic Biology of Stem Cells (LABCET), Instituto Carlos Chagas - FIOCRUZ-PR, Curitiba, Paraná, Rua Prof. Algacyr Munhoz Mader, 3775, CIC, 81830-010, Brazil.

Tel: +55 41 21043231

Email: bernardobonilauri@gmail.com

**Supplementary FigureS1** Principal Component Analysis

FigureS1: Principal Component Analysis for the normalized data. (A) Samples from HIIT, (B) RT, (C) CT and (D) ET.

**Supplementary FigureS2** Coexpression network analysis

FigureS2: Modules of coexpression network analysis. (A) Module 2 with 8 DE lncRNAs in HIIT. (B,C,D) Module 2, 1 and 4 with 3, 1 and 1 DELS in RT, respectively.

**Supplementary Table S1: DE lncRNAs (High-Intensity Interval Training)**

| **gene_name** | **ENSEMBL gene_id** | **log2 (FoldChange)** | **Adjust P-value** |
| --- | --- | --- | --- |
| AC010198.2 | ENSG00000285517.1 | 5,337336 | 0,00269937 |
| AP000688.2 | ENSG00000233393.1 | 4,42899 | 0,002411423 |
| CLLU1 | ENSG00000257127.6 | 4,307085 | 0,06928894 |
| FEZF1-AS1 | ENSG00000230316.7 | 4,0678 | 0,0112147 |
| AC007222.2 | ENSG00000287340.1 | 3,807618 | 7,59E-05 |
| AC099521.2 | ENSG00000279693.1 | 3,796083 | 0,004341135 |
| DLEU2L | ENSG00000116652.6 | 3,642038476 | 8,14E-05 |
| LINC02593 | ENSG00000223764.2 | 3,371082 | 0,02115259 |
| AC087878.1 | ENSG00000259278.2 | 3,204094 | 0,08352431 |
| AL355474.1 | ENSG00000287969.1 | 3,200667 | 0,003482506 |
| AL158195.1 | ENSG00000286191.1 | 3,080102 | 0,000473905 |
| AP002761.3 | ENSG00000257038.1 | 2,941608 | 0,0303898 |
| LINC00163 | ENSG00000234880.1 | 2,87724 | 0,0195786 |
| AL022344.1 | ENSG00000259869.2 | 2,529984 | 0,001356858 |
| PEG13 | ENSG00000282164.3 | 2,447252 | 0,002588579 |
| AC073283.2 | ENSG00000226087.2 | 2,443059 | 0,004027442 |
| AC091182.1 | ENSG00000253123.4 | 2,298192 | 0,01213283 |
| LINC00930 | ENSG00000258647.6 | 2,291004 | 0,004574733 |
| LINC02397 | ENSG00000205056.8 | 2,275081 | 0,02585187 |
| TET2-AS1 | ENSG00000251586.1 | 2,273144 | 0,01322468 |
| TENM3-AS1 | ENSG00000177822.8 | 2,268279 | 0,08379387 |
| MIR503HG | ENSG00000223749.10 | 2,252887 | 0,007730969 |
| LINC00184 | ENSG00000224939.1 | 2,17712 | 0,002937501 |
| AL117335.1 | ENSG00000276649.1 | 2,124271 | 0,04693408 |
| AC023480.1 | ENSG00000287720.1 | 2,060004 | 0,00551539 |
| AC005165.1 | ENSG00000223561.7 | 2,033609 | 0,03589069 |
| AC091576.1 | ENSG00000285681.2 | 1,996581 | 0,08675506 |
| MAPT-AS1 | ENSG00000264589.4 | 1,922244 | 0,02404285 |
| AC020594.1 | ENSG00000237133.1 | 1,900888 | 0,04888999 |
| ARRDC3-AS1 | ENSG00000281357.3 | 1,847837 | 0,04666449 |
| AC106820.4 | ENSG00000260293.2 | 1,832196 | 0,0213953 |
| LINCMD1 | ENSG00000225613.2 | 1,807551 | 0,01686133 |
| AC110611.2 | ENSG00000287382.1 | 1,776503 | 0,01867879 |
| LINC02408 | ENSG00000203585.4 | 1,707738 | 0,03199017 |
| TSPOAP1-AS1 | ENSG00000265148.6 | 1,694089 | 0,01113236 |
| AC090192.2 | ENSG00000253227.2 | 1,685606 | 0,06098815 |
| LINC00840 | ENSG00000226808.3 | 1,684531 | 0,03112791 |
| LINC01176 | ENSG00000281404.2 | 1,680278 | 0,03440167 |
| AC012433.2 | ENSG00000287381.1 | 1,676177 | 0,01433221 |
| AC005329.1 | ENSG00000248015.7 | 1,67299 | 0,027524 |
| AC006511.3 | ENSG00000279865.1 | 1,660646 | 0,04061003 |
| SERPINB9P1 | ENSG00000230438.7 | 1,642882 | 0,04595566 |
| LINC00342 | ENSG00000232931.6 | 1,639395 | 0,007613215 |
| GSEC | ENSG00000280832.1 | 1,638565 | 0,01673815 |
| GAS6-AS1 | ENSG00000233695.2 | 1,624965 | 0,03352927 |
| AC090164.5 | ENSG00000286733.1 | 1,622871 | 0,005198263 |
| RMRP | ENSG00000269900.3 | 1,591895 | 0,02895684 |
| AC010457.1 | ENSG00000251257.2 | 1,583502 | 1,00E-05 |
| AC002525.1 | ENSG00000279314.1 | 1,513007 | 0,02219613 |
| RGMB-AS1 | ENSG00000246763.7 | 1,496271 | 0,01644949 |
| WWC2-AS2 | ENSG00000251359.4 | 1,492244 | 0,07850295 |
| AC083862.3 | ENSG00000287733.1 | 1,45233 | 0,006147065 |
| AC026691.1 | ENSG00000270021.1 | 1,420796 | 0,003005672 |
| AC012636.1 | ENSG00000249825.6 | 1,419099 | 0,004728297 |
| AP001453.2 | ENSG00000256940.2 | 1,389973 | 0,004957782 |
| LINC02009 | ENSG00000283646.2 | 1,389229 | 0,09032266 |
| AL049775.1 | ENSG00000205562.3 | 1,385226 | 0,06629117 |
| LINC00205 | ENSG00000223768.2 | 1,364752 | 0,006553725 |
| NRIR | ENSG00000225964.6 | 1,364676 | 0,03065951 |
| FP236315.1 | ENSG00000279064.1 | 1,363831 | 0,06381264 |
| AC110611.1 | ENSG00000286599.1 | 1,35643 | 0,09085157 |
| AC007881.3 | ENSG00000272735.1 | 1,337766 | 0,03412784 |
| NBAT1 | ENSG00000260455.2 | 1,329301 | 0,09809799 |
| AC023494.1 | ENSG00000286584.1 | 1,31782 | 0,08748184 |
| LINC00565 | ENSG00000260910.1 | 1,31568 | 0,05569346 |
| AC022382.2 | ENSG00000287878.1 | 1,299675 | 0,09975611 |
| AC106820.3 | ENSG00000260095.1 | 1,296631 | 0,02567062 |
| AC007780.1 | ENSG00000267009.6 | 1,28852 | 0,06776247 |
| ID2-AS1 | ENSG00000235092.6 | 1,271579 | 0,00225214 |
| C2orf27A | ENSG00000287151.1 | 1,267839 | 0,01908038 |
| AL354836.1 | ENSG00000226332.2 | 1,247005 | 0,03130823 |
| CASC15 | ENSG00000272168.8 | 1,213838 | 0,00225214 |
| AC024361.2 | ENSG00000263063.1 | 1,187733 | 0,0381854 |
| AC093425.1 | ENSG00000224609.8 | 1,17489 | 0,09227082 |
| TMCO1-AS1 | ENSG00000224358.1 | 1,153568 | 0,08673362 |
| STARD13-AS | ENSG00000236581.9 | 1,151567 | 0,08177954 |
| AC121757.2 | ENSG00000287051.1 | 1,151141 | 0,07841732 |
| MIR4435-2HG | ENSG00000172965.16 | 1,139521 | 0,001408653 |
| RALY-AS1 | ENSG00000285230.2 | 1,134889 | 0,004201289 |
| DIRC3 | ENSG00000231672.7 | 1,128167 | 0,04961777 |
| AC022007.1 | ENSG00000206567.10 | 1,127558 | 0,02074898 |
| AC069224.1 | ENSG00000260572.1 | 1,121755 | 0,03809512 |
| AC079848.2 | ENSG00000287232.1 | 1,113101 | 0,0961009 |
| BX640514.2 | ENSG00000273812.3 | 1,110112 | 0,0618691 |
| AL022328.3 | ENSG00000273188.1 | 1,109065 | 0,09500293 |
| AC011451.1 | ENSG00000267510.1 | 1,085671 | 0,03572629 |
| LINC00963 | ENSG00000204054.14 | 1,084281 | 0,000186985 |
| AC092111.1 | ENSG00000275367.1 | 1,080115 | 0,0657068 |
| AC107204.1 | ENSG00000281392.2 | 1,064163 | 0,09166078 |
| MSC-AS1 | ENSG00000235531.10 | 1,03623 | 0,01323545 |
| AC009041.2 | ENSG00000260807.7 | 1,029485 | 0,05710062 |
| CYTOR | ENSG00000222041.11 | 1,019614 | 0,08778681 |
| MAN1B1-DT | ENSG00000268996.3 | 1,015139 | 0,002327211 |
| SLC9A3-AS1 | ENSG00000225138.8 | 1,008525 | 0,09186216 |
| WNT5A-AS1 | ENSG00000244586.1 | 1,006895 | 0,03893512 |
| KCNQ5-AS1 | ENSG00000229154.2 | 1,005883 | 0,07307816 |
| AC009570.1 | ENSG00000272986.1 | 1,000504 | 0,0838523 |
| AL135910.1 | ENSG00000272848.2 | 0,9921362 | 0,07659188 |
| AC087741.1 | ENSG00000262580.5 | 0,9899354 | 0,03401635 |
| AC093673.1 | ENSG00000232533.1 | 0,9889844 | 0,01789467 |
| TGFB2-AS1 | ENSG00000232480.1 | 0,986445 | 0,032567 |
| ADIRF-AS1 | ENSG00000272734.1 | 0,976989 | 0,02639708 |
| AC008074.2 | ENSG00000260101.1 | 0,9769529 | 0,0992729 |
| AC008555.1 | ENSG00000261754.2 | 0,9696844 | 0,09745732 |
| AL357060.1 | ENSG00000237499.7 | 0,9562512 | 0,06485258 |
| AL035681.1 | ENSG00000235513.2 | 0,9267007 | 0,02251601 |
| FBXL19-AS1 | ENSG00000260852.1 | 0,9163071 | 0,003005672 |
| ITGA9-AS1 | ENSG00000235257.9 | 0,9138404 | 0,08368556 |
| HDAC2-AS2 | ENSG00000228624.7 | 0,8885896 | 0,09991985 |
| AC009271.1 | ENSG00000267327.2 | 0,8879246 | 0,01088769 |
| AC100786.1 | ENSG00000246731.3 | 0,8751456 | 0,05134264 |
| FZD10-AS1 | ENSG00000250208.6 | 0,8552938 | 0,09829739 |
| PAQR9-AS1 | ENSG00000241570.8 | 0,8486059 | 0,09753887 |
| AL133367.1 | ENSG00000260285.1 | 0,8341532 | 0,05842054 |
| COLCA1 | ENSG00000196167.10 | 0,8280826 | 0,09829411 |
| AL118558.4 | ENSG00000272444.1 | 0,8279099 | 0,03050287 |
| CNTFR-AS1 | ENSG00000237159.6 | 0,8092624 | 0,08136712 |
| AL136084.3 | ENSG00000270412.1 | 0,8040378 | 0,05269726 |
| EXTL3-AS1 | ENSG00000246339.6 | 0,8027822 | 0,06657371 |
| AL137077.2 | ENSG00000283078.1 | 0,7984755 | 0,05864482 |
| H19 | ENSG00000130600.19 | 0,792807694 | 0,071781852 |
| AC006059.1 | ENSG00000230084.6 | 0,7877965 | 0,001682385 |
| LINC01547 | ENSG00000183250.12 | 0,7817567 | 0,03211202 |
| LINC00517 | ENSG00000259091.1 | 0,7703716 | 0,05899761 |
| SLC25A25-AS1 | ENSG00000234771.3 | 0,7698582 | 0,004758832 |
| LINC02447 | ENSG00000245468.4 | 0,7626842 | 0,05174377 |
| AC092279.1 | ENSG00000268362.6 | 0,7603272 | 0,09995062 |
| AL139260.1 | ENSG00000228436.3 | 0,7523301 | 0,04398342 |
| AC116407.1 | ENSG00000214708.4 | 0,7508676 | 0,03117035 |
| AC092645.1 | ENSG00000278962.1 | 0,7502185 | 0,05481913 |
| AL161665.2 | ENSG00000286527.1 | 0,7471801 | 0,04964073 |
| AP000873.2 | ENSG00000247137.9 | 0,7395055 | 0,0578995 |
| EIF2AK3-DT | ENSG00000234028.3 | 0,7388442 | 0,08853294 |
| AC007743.1 | ENSG00000233251.8 | 0,7056033 | 0,04432942 |
| RNF144A-AS1 | ENSG00000228203.7 | 0,6980128 | 0,0763706 |
| HCP5 | ENSG00000206337.11 | 0,6637883 | 0,029215 |
| ZNF460-AS1 | ENSG00000267871.6 | 0,6615628 | 0,02889976 |
| PRRT3-AS1 | ENSG00000230082.1 | 0,6561395 | 0,03511331 |
| AL138756.1 | ENSG00000259953.2 | 0,6558145 | 0,06416512 |
| AC008969.1 | ENSG00000176593.8 | 0,6510249 | 0,06829545 |
| AC004812.2 | ENSG00000277283.1 | 0,6482466 | 0,09028218 |
| LAMTOR5-AS1 | ENSG00000224699.9 | 0,6431217 | 0,01866739 |
| ZBTB11-AS1 | ENSG00000256628.3 | 0,6325329 | 0,05635619 |
| HOTAIRM1 | ENSG00000233429.9 | 0,6239491 | 0,09867759 |
| AC016727.1 | ENSG00000270820.6 | 0,6202989 | 0,01322468 |
| AC107375.1 | ENSG00000259891.1 | 0,6143845 | 0,0838523 |
| AC016747.1 | ENSG00000212978.6 | 0,6020163 | 0,05842054 |
| AC072061.1 | ENSG00000259826.1 | 0,6008572 | 0,05241359 |
| BDNF-AS | ENSG00000245573.9 | 0,5932843 | 0,03064115 |
| HOXA-AS3 | ENSG00000254369.6 | 0,5780849 | 0,08450847 |
| RTCA-AS1 | ENSG00000224616.3 | 0,5687709 | 0,06145617 |
| UBA6-AS1 | ENSG00000248049.7 | 0,555664 | 0,01430828 |
| ERVK13-1 | ENSG00000260565.7 | 0,5436477 | 0,04970589 |
| PRKRA-AS1 | ENSG00000223960.7 | 0,5391065 | 0,001578487 |
| AL137782.1 | ENSG00000261553.5 | 0,5164671 | 0,0505982 |
| SREBF2-AS1 | ENSG00000184068.2 | 0,5144079 | 0,0961157 |
| CARMN | ENSG00000249669.10 | 0,508403 | 0,08051633 |
| AC012313.1 | ENSG00000232098.3 | 0,504385 | 0,04767146 |
| AC010226.1 | ENSG00000249249.2 | -0,5003122 | 0,01275055 |
| HOXC-AS1 | ENSG00000250451.5 | -0,5079598 | 0,08260582 |
| AL109936.2 | ENSG00000271420.1 | -0,5303115 | 0,05710062 |
| AC069360.1 | ENSG00000250041.3 | -0,5392151 | 0,000344388 |
| AC097448.1 | ENSG00000288066.1 | -0,5488945 | 0,001977247 |
| AC091152.2 | ENSG00000267160.1 | -0,5541765 | 0,06607178 |
| SNHG5 | ENSG00000203875.12 | -0,5641151 | 0,007693546 |
| AC027575.2 | ENSG00000278107.1 | -0,5668928 | 0,03131356 |
| AC138474.1 | ENSG00000267417.1 | -0,5690181 | 0,01708277 |
| AL158070.2 | ENSG00000279609.1 | -0,6107894 | 0,07453399 |
| AC104564.1 | ENSG00000263370.1 | -0,6269508 | 0,000638959 |
| AC015909.5 | ENSG00000279792.1 | -0,6282416 | 0,07882868 |
| AC006333.1 | ENSG00000230442.5 | -0,6597599 | 0,02895851 |
| AC124016.2 | ENSG00000273156.2 | -0,660176 | 0,0043254 |
| AC092376.3 | ENSG00000285918.1 | -0,67646 | 0,07267767 |
| AL133444.1 | ENSG00000285774.2 | -0,6804995 | 0,08499933 |
| AC084824.5 | ENSG00000276148.1 | -0,700634 | 0,08464741 |
| AL355596.2 | ENSG00000286452.1 | -0,7095052 | 0,000875346 |
| AC011912.1 | ENSG00000279145.2 | -0,7175181 | 0,02397316 |
| AC005632.5 | ENSG00000287809.1 | -0,7195586 | 0,05266047 |
| ALKBH3-AS1 | ENSG00000244926.7 | -0,7350358 | 0,08560243 |
| LINC00390 | ENSG00000226519.2 | -0,7441044 | 0,01322468 |
| AC022532.1 | ENSG00000280401.1 | -0,7727131 | 0,01339111 |
| AC092802.4 | ENSG00000287919.1 | -0,7727945 | 0,09539174 |
| AL445423.1 | ENSG00000272823.1 | -0,7884852 | 0,08368556 |
| PKN2-AS1 | ENSG00000237505.8 | -0,7971422 | 0,05090287 |
| LINC01352 | ENSG00000238078.1 | -0,800367 | 0,0212773 |
| AC108058.1 | ENSG00000238273.3 | -0,8043453 | 0,09588408 |
| HSD11B1-AS1 | ENSG00000227591.5 | -0,8121184 | 0,004016001 |
| Z95331.1 | ENSG00000280383.1 | -0,8418225 | 0,07567319 |
| AC113133.1 | ENSG00000253389.2 | -0,8665831 | 0,005312777 |
| LINC02028 | ENSG00000230102.8 | -0,8783106 | 0,0114819 |
| AC022706.1 | ENSG00000267364.2 | -0,8821306 | 0,04896197 |
| AC016705.2 | ENSG00000259495.3 | -0,8994936 | 0,01097194 |
| CNNM3-DT | ENSG00000273265.1 | -0,9216987 | 0,01465719 |
| LANCL1-AS1 | ENSG00000234281.5 | -0,9444551 | 0,01113136 |
| LINC00310 | ENSG00000227456.9 | -0,9543585 | 0,009909977 |
| AC092376.1 | ENSG00000261722.1 | -0,9764601 | 0,09724726 |
| SMIM2-IT1 | ENSG00000235285.1 | -1,081458 | 0,01014714 |
| AC129803.1 | ENSG00000287378.1 | -1,083918 | 0,04617292 |
| LINC01754 | ENSG00000224565.2 | -1,125128 | 0,04054591 |
| AC131934.1 | ENSG00000255158.1 | -1,412476 | 0,01571136 |
| AC099754.1 | ENSG00000225386.2 | -1,602351 | 0,05119641 |
| AC098936.1 | ENSG00000224717.1 | -1,646567 | 0,04329663 |
| AC010105.1 | ENSG00000237498.3 | -2,222675 | 0,05706161 |
| AC018467.1 | ENSG00000232451.2 | -2,826707 | 6,23E-05 |

**Supplementary Table S2: DE lncRNAs (Resistance training).**

| **gene_name** | **ENSEMBL gene_id** | **log2 (FoldChange)** | **Adjust P-value** |
| --- | --- | --- | --- |
| AC009754.2 | ENSG00000287500.1 | 1,648234149 | 0,02439897 |
| AL009178.2 | ENSG00000269155.2 | 1,460451375 | 0,07680567 |
| AC009041.2 | ENSG00000260807.7 | 1,375101572 | 0,006921323 |
| AC132872.4 | ENSG00000280407.2 | 1,261719565 | 0,083511263 |
| AC012636.1 | ENSG00000249825.6 | 1,225852323 | 0,009093522 |
| AL355377.3 | ENSG00000286782.1 | 1,185081463 | 0,058429206 |
| C22orf34 | ENSG00000188511.12 | 1,119955358 | 0,082952782 |
| AP001453.2 | ENSG00000256940.2 | 1,091222682 | 0,01380389 |
| FAM181A-AS1 | ENSG00000258584.2 | 1,056295836 | 0,035419771 |
| AL096701.4 | ENSG00000287817.1 | 1,024218727 | 0,085247809 |
| AC007743.1 | ENSG00000233251.8 | 1,006827154 | 0,004407752 |
| FAM182B | ENSG00000175170.16 | 0,979133939 | 0,089312104 |
| AL359220.1 | ENSG00000247287.3 | 0,964845303 | 0,01884387 |
| CYTOR | ENSG00000222041.11 | 0,938030835 | 0,068245742 |
| AP003465.1 | ENSG00000254224.2 | 0,915956825 | 0,0329646 |
| AC005856.1 | ENSG00000286997.1 | 0,901552622 | 0,076781687 |
| TBX2-AS1 | ENSG00000267280.5 | 0,8523969 | 0,067667228 |
| AC007406.3 | ENSG00000256540.1 | 0,829330198 | 0,004611689 |
| AC005839.1 | ENSG00000279089.1 | 0,823353127 | 0,048878372 |
| LINC02587 | ENSG00000229108.2 | 0,799524234 | 0,005360002 |
| AL713852.1 | ENSG00000285847.2 | 0,790740349 | 0,028095607 |
| AC026369.2 | ENSG00000256694.1 | 0,776643355 | 0,008710518 |
| AC087289.6 | ENSG00000267801.1 | 0,757752562 | 0,050575671 |
| AL161668.4 | ENSG00000258604.1 | 0,726366874 | 0,018221862 |
| WDFY3-AS2 | ENSG00000180769.10 | 0,682701696 | 0,001440592 |
| AL132642.1 | ENSG00000258987.2 | 0,673863333 | 0,082952782 |
| AC022007.1 | ENSG00000206567.10 | 0,547369096 | 0,060794645 |
| PCAT19 | ENSG00000267107.8 | 0,520879029 | 0,042117096 |
| HSD11B1-AS1 | ENSG00000227591.5 | -0,503887059 | 0,020486214 |
| AC124016.2 | ENSG00000273156.2 | -0,621004783 | 0,015310441 |
| AC091607.2 | ENSG00000287916.1 | -0,622403705 | 0,021311111 |
| AL356019.2 | ENSG00000258768.2 | -0,688929255 | 0,022062065 |
| AC023632.2 | ENSG00000253704.2 | -0,696755481 | 0,075509995 |
| AP003352.1 | ENSG00000245970.2 | -0,781234069 | 0,096011431 |
| LINC01894 | ENSG00000264345.2 | -0,803829599 | 0,077122536 |
| AC006538.2 | ENSG00000261342.1 | -0,890907409 | 0,028425637 |
| PRKCZ-AS1 | ENSG00000182873.5 | -0,891438668 | 0,029526489 |
| AC006059.5 | ENSG00000287629.1 | -0,993430923 | 0,073467732 |
| AC063919.1 | ENSG00000251448.2 | -1,136176909 | 0,073259582 |
| AC073389.2 | ENSG00000271848.2 | -1,200574142 | 0,06719699 |
| AC116535.1 | ENSG00000246308.2 | -1,212939935 | 0,081982148 |
| AC018467.1 | ENSG00000232451.2 | -1,31405652 | 0,068932679 |
| AL137127.1 | ENSG00000272084.1 | -1,476674869 | 0,012155312 |

**Supplementary Table S3: DE lncRNAs (Combined training).**

| **gene_name** | **ENSEMBL gene_id** | **log2 (FoldChange)** | **Adjust P-value** |
| --- | --- | --- | --- |
| AL513318.2 | ENSG00000269994.3 | 3,258826991 | 0,03609946 |
| AL359704.3 | ENSG00000287627.1 | 1,464160721 | 0,018942561 |
| AL136366.1 | ENSG00000225472.2 | 1,027844846 | 0,016146188 |
| AC015878.1 | ENSG00000265751.1 | 0,885386586 | 0,03609946 |
| USP2-AS1 | ENSG00000245248.8 | 0,864120876 | 0,052164916 |
| LINC02587 | ENSG00000229108.2 | 0,789323557 | 0,048133258 |
| AC090004.2 | ENSG00000287059.1 | 0,684476529 | 0,09255423 |
| AC027682.6 | ENSG00000276075.1 | 0,65694129 | 0,097572802 |
| AC104083.1 | ENSG00000260244.1 | 0,588891395 | 0,097920749 |
| HCP5 | ENSG00000206337.11 | 0,514202112 | 0,026820467 |
| LINC-PINT | ENSG00000231721.7 | -0,579389735 | 0,09306066 |
| AL662844.4 | ENSG00000272501.1 | -0,645766658 | 0,087002078 |
| AC022706.1 | ENSG00000267364.2 | -0,674883292 | 0,075929094 |
| LINC01091 | ENSG00000249464.6 | -0,796805593 | 0,069644818 |
| AC012378.2 | ENSG00000285805.1 | -0,838777521 | 0,0772143 |

**Supplementary Table S4: DE lncRNAs (Endurance Training).**

| **gene_name** | **ENSEMBL gene_id** | **log2 (FoldChange)** | **Adjust P-value** |
| --- | --- | --- | --- |
| AL009178.2 | ENSG00000269155.2 | 2,4056036 | 0,000522227 |
| MIR503HG | ENSG00000223749.10 | 2,008939267 | 0,070210208 |
| AC090164.5 | ENSG00000286733.1 | 1,991275111 | 5,49E-05 |
| AL450043.1 | ENSG00000285802.1 | 1,87036926 | 0,079308596 |
| AC012636.1 | ENSG00000249825.6 | 1,610191762 | 0,007118999 |
| AC010457.1 | ENSG00000251257.2 | 1,466322843 | 0,032840663 |
| CYTOR | ENSG00000222041.11 | 1,375485813 | 0,025042324 |
| CASC15 | ENSG00000272168.8 | 1,357271246 | 0,012368229 |
| ID2-AS1 | ENSG00000235092.6 | 1,268761335 | 0,092663309 |
| SERPINB9P1 | ENSG00000230438.7 | 1,136741993 | 0,054400963 |
| LINC00639 | ENSG00000259070.7 | 1,135354189 | 0,079069386 |
| HAGLR | ENSG00000224189.8 | 1,066139332 | 0,091649729 |
| AL513534.2 | ENSG00000260400.1 | 0,987085385 | 0,0828647 |
| AC015878.1 | ENSG00000265751.1 | 0,984501745 | 0,004602652 |
| HCP5 | ENSG00000206337.11 | 0,91649734 | 0,00283607 |
| LINC02587 | ENSG00000229108.2 | 0,909995905 | 0,022396871 |
| AL138781.2 | ENSG00000275329.1 | 0,900032536 | 0,05990599 |
| H19 | ENSG00000130600.19 | 0,86991241 | 0,045163651 |
| LINC00963 | ENSG00000204054.14 | 0,835715743 | 0,031367451 |
| AC100803.1 | ENSG00000244998.1 | 0,763725761 | 0,034800316 |
| AL118506.1 | ENSG00000268858.2 | 0,630691576 | 0,076112356 |
| CARD8-AS1 | ENSG00000268001.1 | 0,538355471 | 0,091924975 |
| SNHG6 | ENSG00000245910.8 | -0,534148637 | 0,020207348 |
| AC068700.1 | ENSG00000260398.1 | -0,542941877 | 0,000134157 |
| C10orf71-AS1 | ENSG00000236208.1 | -0,546230969 | 0,079636799 |
| ACVR2B-AS1 | ENSG00000229589.1 | -0,557906168 | 0,050786689 |
| PRR34-AS1 | ENSG00000241990.5 | -0,575725262 | 0,036858903 |
| LINC02541 | ENSG00000230943.3 | -0,59283899 | 0,044556766 |
| LANCL1-AS1 | ENSG00000234281.5 | -0,598503275 | 0,034850695 |
| AC097448.1 | ENSG00000288066.1 | -0,600195249 | 0,073267764 |
| MBNL1-AS1 | ENSG00000229619.4 | -0,606377141 | 0,062696324 |
| SOX9-AS1 | ENSG00000234899.10 | -0,625470323 | 0,074481267 |
| AC106791.1 | ENSG00000250159.7 | -0,726894521 | 0,046253344 |
| AC069360.1 | ENSG00000250041.3 | -0,731800557 | 0,000595268 |
| AP003469.4 | ENSG00000261087.1 | -0,762228159 | 0,002016533 |
| AC044839.2 | ENSG00000254519.5 | -0,803103912 | 0,009836803 |
| NEAT1 | ENSG00000245532.9 | -0,810594427 | 0,01863177 |
| AC062015.1 | ENSG00000235070.3 | -0,853466171 | 0,010364987 |
| AC005616.1 | ENSG00000267423.1 | -0,899413192 | 0,006598049 |
| DIO3OS | ENSG00000258498.8 | -0,901108963 | 0,004845737 |
| AC124016.2 | ENSG00000273156.2 | -0,929575497 | 0,003559302 |
| LINC01854 | ENSG00000204460.3 | -0,93785005 | 0,094222226 |
| AC104564.1 | ENSG00000263370.1 | -0,951159468 | 0,022543974 |
| LINC00310 | ENSG00000227456.9 | -1,143797528 | 0,07553561 |
| AC106897.1 | ENSG00000249679.2 | -1,193837538 | 0,00313103 |
| FLNC-AS1 | ENSG00000242902.1 | -1,277728699 | 0,00897262 |
| AC113133.1 | ENSG00000253389.2 | -1,285575436 | 0,000462771 |
| LINC01954 | ENSG00000271952.2 | -1,389547224 | 0,080779978 |
| AC113167.1 | ENSG00000214942.6 | -1,863333895 | 0,061622684 |
| AJ009632.2 | ENSG00000229425.3 | -2,003353808 | 0,076380546 |
| AC099066.2 | ENSG00000227496.3 | -2,380123028 | 1,38E-07 |
| AC018467.1 | ENSG00000232451.2 | -2,587740148 | 0,005129136 |

**Supplementary Table S5: Top10 Gene Ontology Terms of DEGs (protein-coding genes) after HIIT.**

| GO Term | P-Value |
| --- | --- |
| extracellular matrix organization (GO:0030198) | 4,38E-24 |
| collagen fibril organization (GO:0030199) | 7,58E-08 |
| protein complex subunit organization (GO:0071822) | 2,23E-06 |
| extracellular matrix disassembly (GO:0022617) | 3,81E-04 |
| skeletal system development (GO:0001501) | 6,57E-04 |
| platelet degranulation (GO:0002576) | 0.000001213 |
| glycosaminoglycan biosynthetic process (GO:0006024) | 0.000007260 |
| positive regulation of intracellular signal transduction (GO:1902533) | 0.00001061 |
| regulation of angiogenesis (GO:0045765) | 0.00001179 |
| regulated exocytosis (GO:0045055) | 0.00001385 |

**Supplementary Table S6: Top10 Gene Ontology Terms of DEGs (protein-coding genes) after RT.**

| GO Term | P-Value |
| --- | --- |
| extracellular matrix organization (GO:0030198) | 1,75E-14 |
| collagen fibril organization (GO:0030199) | 6,42E-07 |
| regulation of angiogenesis (GO:0045765) | 6,95E-06 |
| regulation of cell migration (GO:0030334) | 1,75E-05 |
| positive regulation of intracellular signal transduction (GO:1902533) | 3,61E-05 |
| protein complex subunit organization (GO:0071822) | 4,50E-05 |
| positive regulation of phosphatidylinositol 3-kinase signaling (GO:0014068) | 2,00E-04 |
| regulation of MAPK cascade (GO:0043408) | 3,05E-04 |
| regulation of phosphatidylinositol 3-kinase signaling (GO:0014066) | 8,17E-04 |
| negative regulation of response to stimulus (GO:0048585) | 0.000001401 |

**Supplementary Table S7: Top10 Gene Ontology Terms of DEGs (protein-coding genes) after CT.**

| GO Term | P-Value |
| --- | --- |
| mitochondrial electron transport, cytochrome c to oxygen (GO:0006123) | 0.000001564 |
| regulation of blood vessel endothelial cell migration (GO:0043535) | 0.00005491 |
| cellular response to peptide hormone stimulus (GO:0071375) | 0.00009480 |
| positive regulation of sprouting angiogenesis (GO:1903672) | 0.0002095 |
| negative regulation of endothelial cell proliferation (GO:0001937) | 0.0002580 |
| regulation of endothelial cell proliferation (GO:0001936) | 0.0003270 |
| positive regulation of blood vessel endothelial cell proliferation involved in sprouting angiogenesis (GO:1903589) | 0.0003525 |
| extracellular matrix organization (GO:0030198) | 0.0003821 |
| mitochondrial ATP synthesis coupled electron transport (GO:0042775) | 0.0004324 |
| basement membrane organization (GO:0071711) | 0.0004688 |

**Supplementary Table S8: Top10 Gene Ontology Terms of DEGs (protein-coding genes) after ET.**

| GO Term | P-Value |
| --- | --- |
| extracellular matrix organization (GO:0030198) | 8,39E-25 |
| endoderm formation (GO:0001706) | 2,93E-08 |
| collagen fibril organization (GO:0030199) | 3,88E-08 |
| regulation of angiogenesis (GO:0045765) | 6,87E-08 |
| protein complex subunit organization (GO:0071822) | 5,52E-07 |
| regulation of cell migration (GO:0030334) | 1,14E-06 |
| endodermal cell differentiation (GO:0035987) | 2,77E-06 |
| sprouting angiogenesis (GO:0002040) | 6,18E-06 |
| extracellular matrix disassembly (GO:0022617) | 4,93E-05 |

**Supplementary Table S9: Module1 DE lncRNAs (High-Intensity Interval Training).**

| **gene_name** | **ENSEMBL gene_id** | **log2 (FoldChange)** | **Adjust P-value** |
| --- | --- | --- | --- |
| AC010198.2 | ENSG00000285517.1 | 5,337336 | 0,00269937 |
| AP000688.2 | ENSG00000233393.1 | 4,42899 | 0,002411423 |
| CLLU1 | ENSG00000257127.6 | 4,307085 | 0,06928894 |
| FEZF1-AS1 | ENSG00000230316.7 | 4,0678 | 0,0112147 |
| AC007222.1 | ENSG00000287340.1 | 3,807618 | 7,59E-05 |
| AC099521.2 | ENSG00000279693.1 | 3,796083 | 0,004341135 |
| DLEU2L | ENSG00000116652.6 | 3,642038476 | 8,14E-05 |
| LINC02593 | ENSG00000223764.2 | 3,371082 | 0,02115259 |
| AC087878.1 | ENSG00000259278.2 | 3,204094 | 0,08352431 |
| AL355474.1 | ENSG00000287969.1 | 3,200667 | 0,003482506 |
| AL158195.1 | ENSG00000286191.1 | 3,080102 | 0,000473905 |
| AP002761.3 | ENSG00000257038.1 | 2,941608 | 0,0303898 |
| LINC00163 | ENSG00000234880.1 | 2,87724 | 0,0195786 |
| AL022344.1 | ENSG00000259869.2 | 2,529984 | 0,001356858 |
| PEG13 | ENSG00000282164.3 | 2,447252 | 0,002588579 |
| AC073283.2 | ENSG00000226087.2 | 2,443059 | 0,004027442 |
| AC091182.1 | ENSG00000253123.4 | 2,298192 | 0,01213283 |
| LINC00930 | ENSG00000258647.6 | 2,291004 | 0,004574733 |
| LINC02397 | ENSG00000205056.8 | 2,275081 | 0,02585187 |
| TET2-AS1 | ENSG00000251586.1 | 2,273144 | 0,01322468 |
| TENM3-AS1 | ENSG00000177822.8 | 2,268279 | 0,08379387 |
| MIR503HG | ENSG00000223749.10 | 2,252887 | 0,007730969 |
| LINC00184 | ENSG00000224939.1 | 2,17712 | 0,002937501 |
| AL117335.1 | ENSG00000276649.1 | 2,124271 | 0,04693408 |
| AC023480.1 | ENSG00000287720.1 | 2,060004 | 0,00551539 |
| AC005165.1 | ENSG00000223561.7 | 2,033609 | 0,03589069 |
| AC091576.1 | ENSG00000285681.2 | 1,996581 | 0,08675506 |
| MAPT-AS1 | ENSG00000264589.4 | 1,922244 | 0,02404285 |
| AC020594.1 | ENSG00000237133.1 | 1,900888 | 0,04888999 |
| ARRDC3-AS1 | ENSG00000281357.3 | 1,847837 | 0,04666449 |
| AC106820.4 | ENSG00000260293.2 | 1,832196 | 0,0213953 |
| LINCMD1 | ENSG00000225613.2 | 1,807551 | 0,01686133 |
| AC110611.2 | ENSG00000287382.1 | 1,776503 | 0,01867879 |
| LINC02408 | ENSG00000203585.4 | 1,707738 | 0,03199017 |
| TSPOAP1-AS1 | ENSG00000265148.6 | 1,694089 | 0,01113236 |
| AC090192.2 | ENSG00000253227.2 | 1,685606 | 0,06098815 |
| LINC00840 | ENSG00000226808.3 | 1,684531 | 0,03112791 |
| LINC01176 | ENSG00000281404.2 | 1,680278 | 0,03440167 |
| AC012433.2 | ENSG00000287381.1 | 1,676177 | 0,01433221 |
| AC005329.1 | ENSG00000248015.7 | 1,67299 | 0,027524 |
| AC006511.3 | ENSG00000279865.1 | 1,660646 | 0,04061003 |
| SERPINB9P1 | ENSG00000230438.7 | 1,642882 | 0,04595566 |
| LINC00342 | ENSG00000232931.6 | 1,639395 | 0,007613215 |
| GAS6-AS1 | ENSG00000233695.2 | 1,624965 | 0,03352927 |
| RMRP | ENSG00000269900.3 | 1,591895 | 0,02895684 |
| AC010457.1 | ENSG00000251257.2 | 1,583502 | 1,00E-05 |
| RGMB-AS1 | ENSG00000246763.7 | 1,496271 | 0,01644949 |
| WWC2-AS2 | ENSG00000251359.4 | 1,492244 | 0,07850295 |
| AC083862.3 | ENSG00000287733.1 | 1,45233 | 0,006147065 |
| AC026691.1 | ENSG00000270021.1 | 1,420796 | 0,003005672 |
| AC012636.1 | ENSG00000249825.6 | 1,419099 | 0,004728297 |
| AP001453.2 | ENSG00000256940.2 | 1,389973 | 0,004957782 |
| LINC02009 | ENSG00000283646.2 | 1,389229 | 0,09032266 |
| AL049775.1 | ENSG00000205562.3 | 1,385226 | 0,06629117 |
| LINC00205 | ENSG00000223768.2 | 1,364752 | 0,006553725 |
| NBAT1 | ENSG00000260455.2 | 1,329301 | 0,09809799 |
| CASC15 | ENSG00000272168.8 | 1,213838 | 0,00225214 |
| AC093425.1 | ENSG00000224609.8 | 1,17489 | 0,09227082 |
| TMCO1-AS1 | ENSG00000224358.1 | 1,153568 | 0,08673362 |
| AC121757.2 | ENSG00000287051.1 | 1,151141 | 0,07841732 |
| DIRC3 | ENSG00000231672.7 | 1,128167 | 0,04961777 |
| AC069224.1 | ENSG00000260572.1 | 1,121755 | 0,03809512 |
| BX640514.2 | ENSG00000273812.3 | 1,110112 | 0,0618691 |
| AC107204.1 | ENSG00000281392.2 | 1,064163 | 0,09166078 |
| AC009041.2 | ENSG00000260807.7 | 1,029485 | 0,05710062 |
| CYTOR | ENSG00000222041.11 | 1,019614 | 0,08778681 |
| SLC9A3-AS1 | ENSG00000225138.8 | 1,008525 | 0,09186216 |
| AL135910.1 | ENSG00000272848.2 | 0,9921362 | 0,07659188 |
| ADIRF-AS1 | ENSG00000272734.1 | 0,976989 | 0,02639708 |
| AL357060.1 | ENSG00000237499.7 | 0,9562512 | 0,06485258 |
| ITGA9-AS1 | ENSG00000235257.9 | 0,9138404 | 0,08368556 |
| AC098936.1 | ENSG00000224717.1 | -1,646567 | 0,04329663 |

**Supplementary Table S10: Module3 DE lncRNAs (Resistance training).**

| **gene_name** | **ENSEMBL gene_id** | **log2 (FoldChange)** | **Adjust P-value** |
| --- | --- | --- | --- |
| AC007743.1 | ENSG00000233251.8 | 1,006827154 | 0,004407752 |
| AC009754.2 | ENSG00000287500.1 | 1,648234149 | 0,02439897 |
| AC018467.1 | ENSG00000232451.2 | -1,31405652 | 0,068932679 |
| AL009178.2 | ENSG00000269155.2 | 1,460451375 | 0,07680567 |
