# Supplementary figures and images for: Long Non-coding RNAs Are Differentially Expressed After Different Exercise Training Programs
Source: Front Physiol. 2020 Sep 15;11:567614. doi: 10.3389/fphys.2020.567614 (PMC7533564; doi:10.3389/fphys.2020.567614)

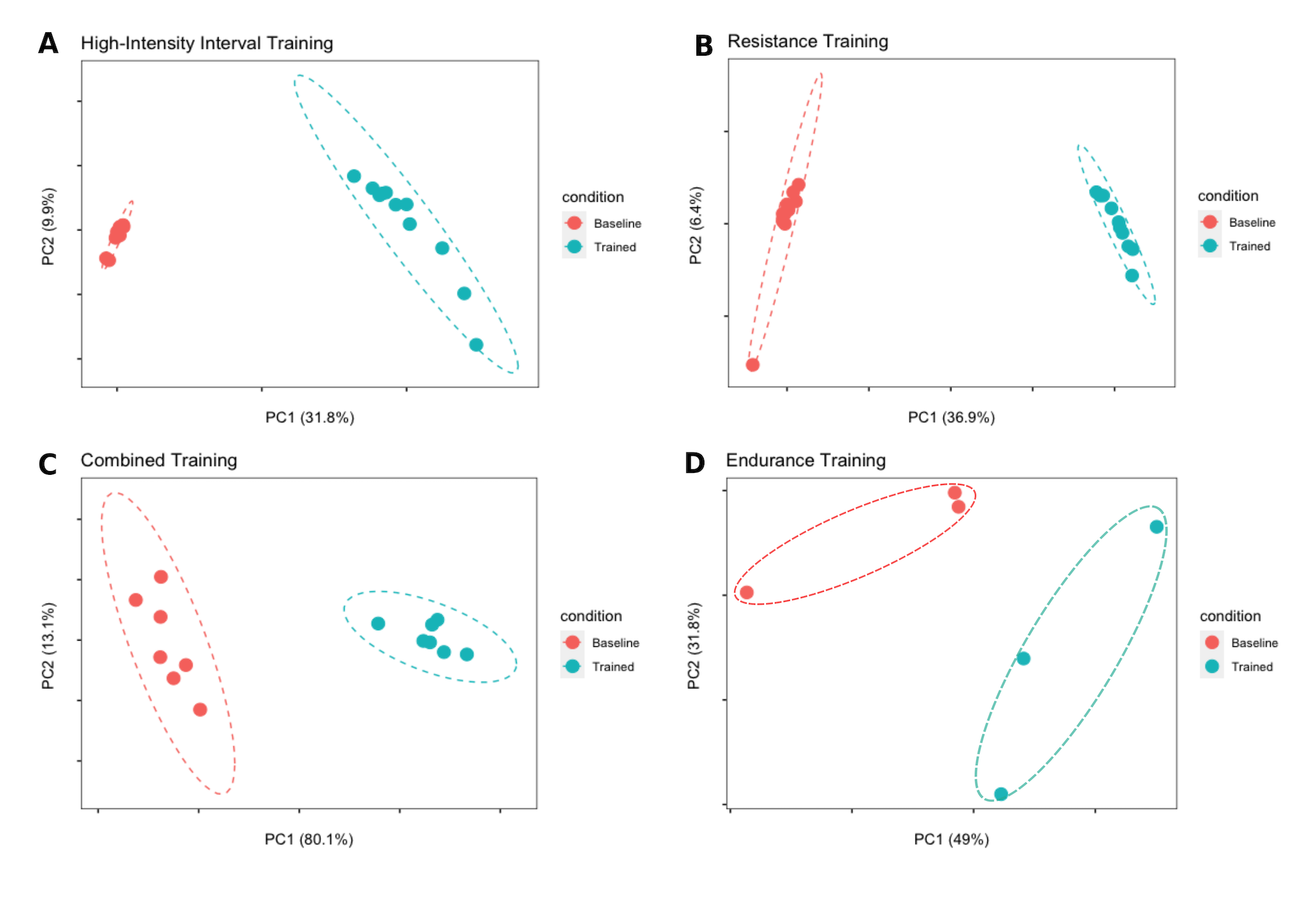

Supplement: Supplementary file 2 [file Image_1.TIFF]

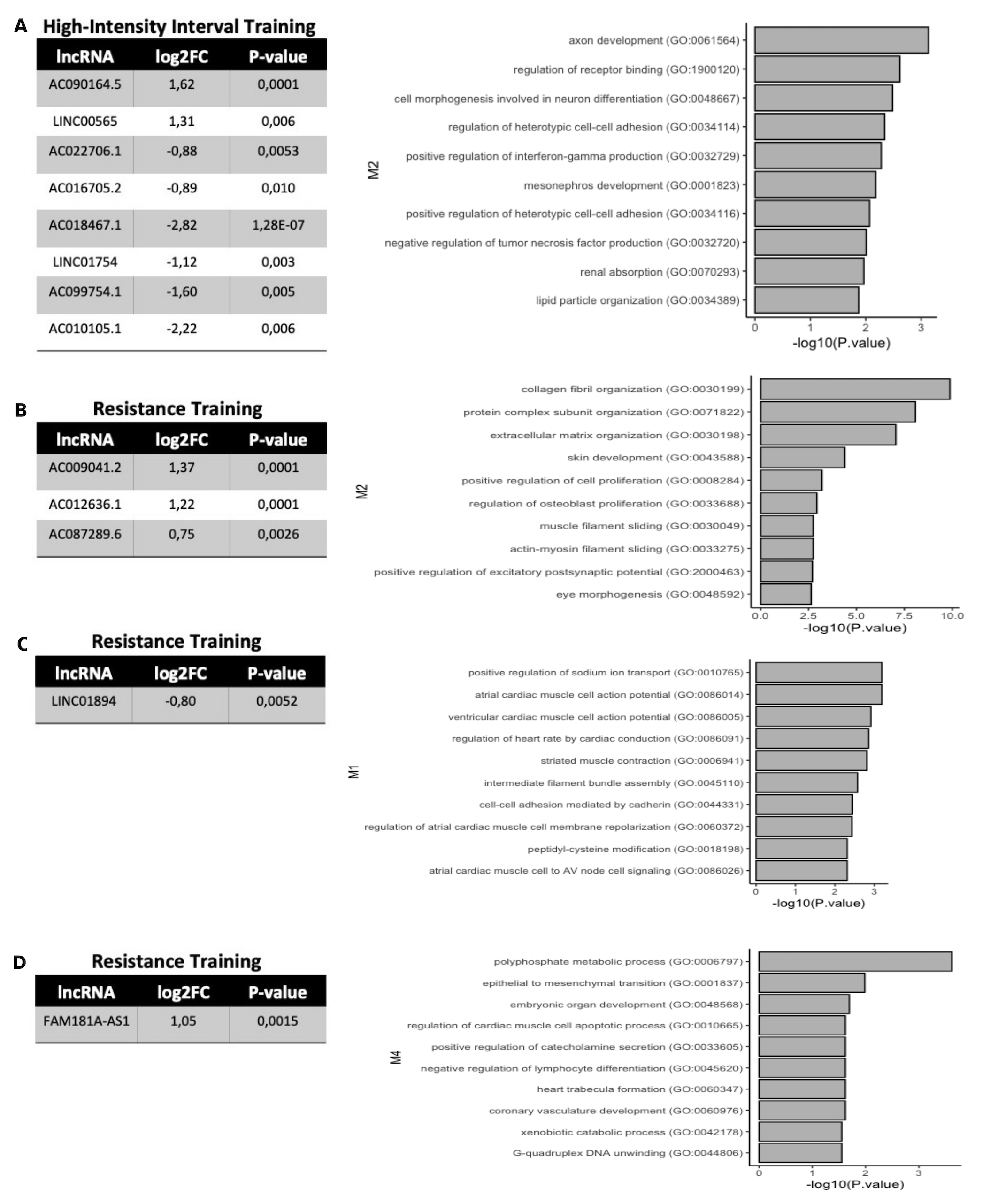

Supplement: Supplementary file 3 [file Image_2.TIFF]
